# Supplementary material for: Hierarchical temporal receptive windows and zero-shot timescale generalization in biologically constrained scale-invariant deep networks
Source: ArXiv. 2026 Jan 6:arXiv:2601.02618v1. Preprint. [Version 1] (PMC12803329)
Supplement: Supplement 1 [file NIHPP2601.02618v1-supplement-1.pdf]

Supporting Information (SI)

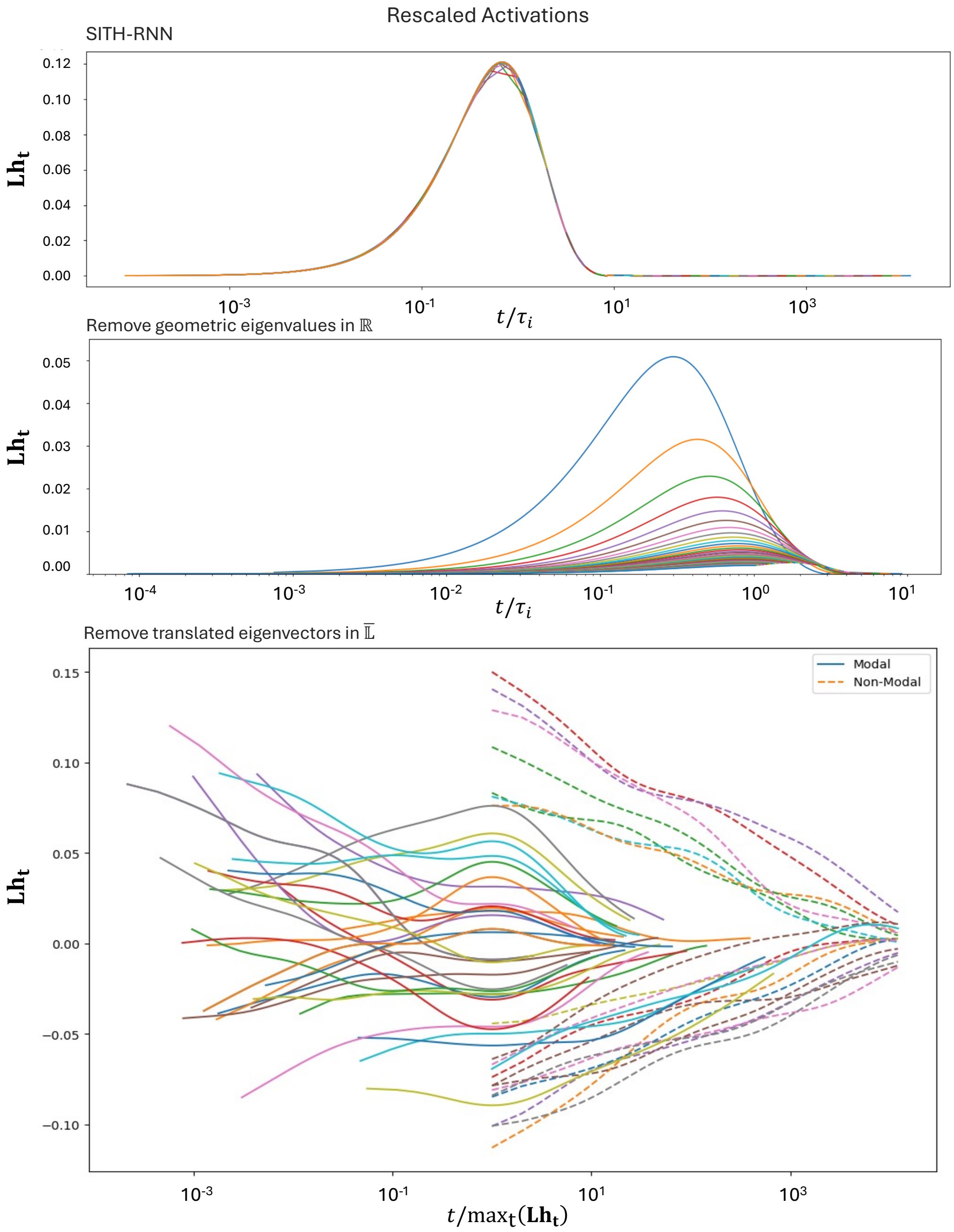

Fig. S1. Ablation study confirms that scale-invariance requires the conjunction of geometric eigenvalues and translation-equivariant readouts. We analyzed the impulse response of the output population $\mathbf{Lh}_t$ to a delta input at $t = 0$ across three model variations. *Top (SITH-RNN):* The full model, featuring both geometric eigenvalues in $\mathbf{R}$ and banded, translation-equivariant readouts in $\mathbf{L}$. When plotted against rescaled time (t/τ_i), the response curves of different neurons collapse onto a single, universal function, indicating true scale-invariance. *Middle (Uniform Eigenvalues):* Replacing the geometric distribution in $\mathbf{R}$ with a uniform distribution preserves sequentiality but destroys scale-invariance. The curves fail to align when rescaled, confirming that logarithmic compression is mathematically necessary for the scaling property. *Bottom (Dense Readout):* Preserving geometric eigenvalues but replacing the banded $\mathbf{L}$ with a standard dense, trainable matrix results in disordered dynamics. The population splits into "Modal" (peaked) and "Non-Modal" (monotonic) responses, losing the interpretable "time cell"-like sequences entirely. Thus, both the geometric eigenvalues in $\mathbf{R}_{when}$ and translated motifs in $\mathbf{L}_{when}$ are required in SITH-RNN to produce scale-invariant, sequential dynamics.

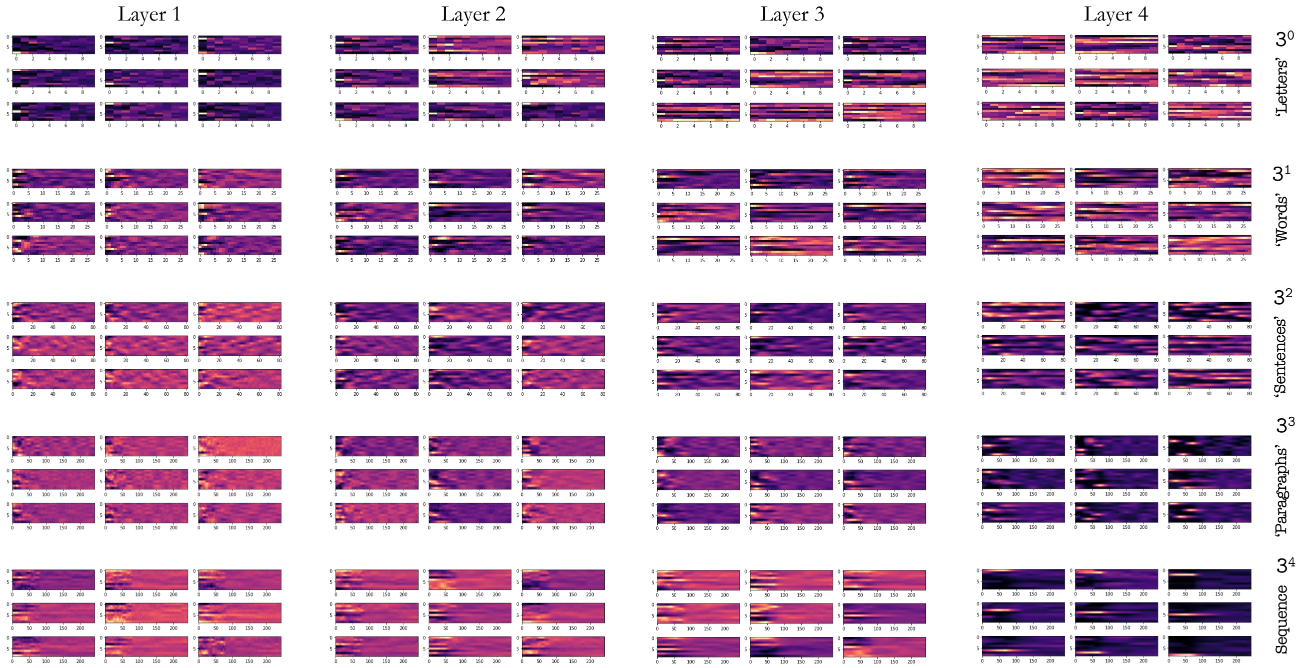

Fig. S2. A comprehensive mapping of linear receptive fields via Spike-Triggered Averaging reveals layer-wise renormalization and learned compositional hierarchy. This figure expands upon the representative examples in Figure 4d by visualizing the linear receptive fields for the complete population of nine neurons across all four hidden layers. Receptive fields were recovered using Spike-Triggered Averaging (STA) relative to the onset of symbols at five hierarchical scales, ranging from elementary *'letters'* (3^0) to full *'sequences'* (3^4). The visualization highlights a fundamental transformation in temporal processing depth. *Left:* Layer 1 neurons display sharp, diagonal banding at the finest timescales, indicating they function as precise detectors for local symbolic transitions—but fail to integrate global context. At higher levels (e.g., *'sequence'* or *'paragraphs'*), their receptive fields appear as dense, repetitive tiling patterns—showing a periodicity which indicates that early layers effectively "reset" with each local symbol transition, tracking recurring constituent elements (e.g., every instance of a specific letter triplet) without the capacity to distinguish their unique position within the broader narrative structure. *Right:* In contrast, Layer 4 neurons (right column) exhibit a nested structure that mirrors the compositional rules of the grammar. Proceeding from the bottom to the top of the Layer 4 column, a single broad activation band at the *'sequence'* level resolves into three distinct bands at the *'paragraph'* level, which further subdivide into nine bands at the *'sentence'* level. This fractal-like fractionation confirms that deep neurons have successfully learned the mapping between hierarchical levels, defining their receptive fields through the appropriate combinatorics of constituent symbols (e.g., a specific sequence is recognized as a composition of three specific paragraphs). *Middle:* The transition between these extremes is not binary. The intermediate columns (Layers 2 and 3) exhibit a rich, continuous gradient of integration windows, which demonstrate the network's capacity for mixed selectivity, processing intermediate linguistic structures (e.g., phrases) that exist between the speed of a single symbol and the duration of a full narrative.
